# Mechanisms Linking Red Blood Cell Disorders and Cardiovascular Diseases

**DOI:** 10.1155/2015/682054

**Published:** 2015-02-01

**Authors:** Ioana Mozos

**Affiliations:** Department of Functional Sciences, “Victor Babes” University of Medicine and Pharmacy, T. Vladimirescu Street 14, 300173 Timisoara, Romania

## Abstract

The present paper aims to review the main pathophysiological links between red blood cell disorders and cardiovascular diseases, provides a brief description of the latest studies in this area, and considers implications for clinical practice and therapy. Anemia is associated with a special risk in proatherosclerotic conditions and heart disease and became a new therapeutic target. Guidelines must be updated for the management of patients with red blood cell disorders and cardiovascular diseases, and targets for hemoglobin level should be established. Risk scores in several cardiovascular diseases should include red blood cell count and RDW. Complete blood count and hemorheological parameters represent useful, inexpensive, widely available tools for the management and prognosis of patients with coronary heart disease, heart failure, hypertension, arrhythmias, and stroke. Hypoxia and iron accumulation cause the most important cardiovascular effects of sickle cell disease and thalassemia. Patients with congenital chronic hemolytic anemia undergoing splenectomy should be monitored, considering thromboembolic and cardiovascular risk.

## 1. Introduction

There are several criteria enabling the diagnosis of anemia. Hemoglobin below 13 g/dL and 12 g/dL in men and women, respectively, according to the criteria of the World Health Organization defines anemia.

Anemia, a condition frequently associated with chronic diseases, is an independent risk factor for cardiovascular complications [1] and a 1 g/dL decrease in hemoglobin level is an independent risk factor for cardiac morbidity and mortality [2]. On the other hand, there are several forms of congenital hemolytic anemia with cardiovascular complications.

The present paper aims to review the main pathophysiological links between red blood cell disorders and cardiovascular diseases, provides a brief description of the latest studies in this area, and considers implications for clinical practice and therapy. The present review will enable updating of the guidelines for the management of patients with both red cell disorders and cardiovascular pathology.

## 2. Anemia in Cardiovascular Disease

Multimorbidity is common in patients with cardiovascular diseases [1]. Prognostic markers are needed to identify patients with cardiovascular disease at high risk for adverse events [3]. Several epidemiological studies investigated possible associations between hemorheological profile and cardiovascular disease; hemorheological alterations may be the cause of the disorder, but they may also result from poor tissue perfusion [4]. Hemorheology is the ability of blood to deform and depends on the hematological characteristics able to influence blood flow independently of the vascular wall, including plasma viscosity, hematocrit, erythrocyte aggregation, and deformation [4]. Increased white blood cell count together with elevated plasma fibrinogen levels and hematocrit increases the resistance to blood flow [5].

Anemia causes hypoxia due to decreased hemoglobin level, and there are several nonhemodynamic (increased erythropoietin production, decreased affinity of hemoglobin for oxygen due to an increase in 2,3-diphosphoglycerate) and hemodynamic compensatory mechanisms [6]. The clinical and hemodinamical changes due to acute, short-lasting anemia are reversible, but chronic anemia leads to progressive cardiac enlargement and left ventricular hypertrophy due to volume overload [6].* Cardiovascular compensatory consequences of anemia* include tachycardia, increased cardiac output, a hyperdynamic state due to reduced blood viscosity, and vasodilation enabling tissue perfusion. Arterial dilatation involves also the recruitment of new vessels and formation of collaterals and arteriovenous shunts [7], hypoxic vasodilation due to hypoxia-generated metabolites, flow-mediated vasodilatation, and endothelium-derived relaxing factor [8]. Anemia increases cardiac output, may lead to eccentric left ventricular hypertrophy, activation of the sympathetic nervous system, and stimulation of the renin angiotensin aldosterone system, and is closely associated with chronic inflammation and increased oxidative stress [9]. Increased left ventricular performance results from preload elevation (Frank-Starling mechanism) and increased inotropic state related to sympathetic activity [10, 11]. Tissue hypoxia and changes in blood flow patterns due to low hemoglobin may play an atherogenic role. Cardiovascular complications of anemia are due to worsening of the hyperdynamic state, volume overload, cardiac dilation, valvular failure, and heart failure with increased cardiac output. Resting cardiac output increases only when hemoglobin concentration declines to 10 g/dL or less [6].

Anemia increases morbidity and mortality in cardiovascular diseases, due to compensatory consequences of hypoxia, such as a hyperdynamic state with increased cardiac output, left ventricular hypertrophy and progressive cardiac enlargement, and, probably, a proatherogenic role.

### 2.1. Heart Failure

Congestive heart failure is uncommon in patients with anemia without heart disease and may occur only in cases of severe anemia with hemoglobin of 5 g/dL or less [6]. Anemia is a common comorbidity in patients with* chronic heart failure* and is associated with an increased all-cause and cardiovascular mortality, reduced exercise capacity due to reduced oxygen carrying and storage capacity, impaired quality of life, a higher risk for hospitalization [12, 13], female gender, older age, edema, low body mass index, increased level of neurohormones, a proinflammatory state (elevated C reactive protein and cytokines), and more comorbidities, including hypertension, atrial fibrillation, diabetes mellitus, and chronic renal failure [14]. The increased mortality is due to comorbidities. The prevalence of anemia in heart failure patients varies depending on the type and severity of anemia [1, 13]. The reported prevalence variability (4–61%) is due to lack of consensus on the definition of anemia [13, 15] and due to different exclusion criteria. Anemia is also prevalent in chronic heart failure with preserved ejection fraction [16, 17].

Anemia in patients with heart failure is often normochromic and normocytic, with a low reticulocyte count [14, 18]. Vitamin B12, folic acid, and iron deficiency may also cause anemia in heart failure patients. Deficiencies in vitamin B12 or folic acid may cause megaloblastic anemia. The cause of vitamin B12 deficiency is seldom dietary; it is more likely due to gastrectomy or comorbidities affecting the terminal ileum. Folic acid deficiency is caused by abnormalities in food intake, chronic alcohol abuse, parenteral nutrition, and diseases of the small intestine [14]. Nutritional iron deficiencies caused by anorexia, insufficient diet supply, gastrointestinal malabsorption, and aspirin-induced gastrointestinal bleeding can cause iron deficiency [19–21]. Ferritin is an acute-phase protein, and a reduction in ferritin because of iron deficiency may be masked by an acute inflammatory response in some patients. Renal dysfunction, neurohormonal activation, and proinflammatory cytokines in heart failure enable the development of anemia of chronic disease, with defective iron utilization, inappropriate erythropoietin production, and depressed bone marrow function [19]. Impaired proliferation, differentiation, mobilization, and iron incorporation in hematopoietic stem cells contribute to the bone marrow dysfunction [22]. The decreased renal perfusion in heart failure patients causes renal hypoxia and enables the release of erythropoietin (EPO), but the response of the bone marrow to EPO is blunted due to the proinflammatory cytokines. The activation of the renin-angiotensin-aldosterone system due to the decreased renal perfusion releases angiotensin II, which also stimulates EPO production and bone marrow erythroid progenitor cells [23]. Iron available for erythropoiesis is reduced due to increased proinflammatory cytokines (functional iron deficiency), which decrease ferroportin (release of iron from macrophages) and increase hepcidin (which blocks duodenal iron absorption) and the divalent metal transporter (able to bind and transport divalent metals along the plasmatic membranes) [19]. Proinflammatory cytokines, including tumor necrosis factor and interleukin 6, are not only increased in heart failure, but also inversely related to hemoglobin [24]. Hemodilution has also a contribution to anemia in heart failure patients. Anemia reduces blood viscosity, decreasing systemic vascular resistance due to enhanced nitric oxide-mediated vasodilation. Low blood pressure causes neurohormonal activation, with increased sympathetic and renin-angiotensin-aldosterone activity, impairing renal perfusion and expanding the extracellular space [19]. Volume expansion and vasodilation increase cardiac output and oxygen transport [25, 26]. These mechanisms suggest that correction of anemia is unlikely to improve left ventricular function [19]. The use of angiotensin-converting inhibitors or angiotensin receptor blockers may inhibit EPO synthesis [20] and prevent the breakdown of hematopoiesis inhibitor N-acetyl-seryl-aspartyl-lysyl-proline [27].

Anemia impairs prognosis in heart failure patients due to a reduced oxygen supply, ventricular remodeling, the neurohumoral profile, the proinflammatory state, and several comorbidities, including renal failure and cardiac cachexia. Probably, anemia is both a mediator and a marker of a poor outcome in heart failure [19].

Therapy includes iron, folic acid, blood transfusions, and erythropoiesis-stimulating agents (ESA). ESA may be considered as adjunctive therapy in patients with heart failure, improving, besides hemoglobin level, also left ventricular ejection fraction and functional class and reducing diuretic doses and left ventricular remodeling [28–32]. EPO also confers antiapoptotic effects, required for the survival of myocardial cells after ischemia [33]. On the other hand, erythropoiesis-stimulating agents may increase the risk of cardiovascular events according to several trials [34, 35] due to impaired nitric oxide production and release [36] and the prothrombotic and antifibrinolytic effect. Iron therapy, reported to improve anemia and cardiac function in several studies, increases oxidative stress [37, 38]. Iron application is controversially debated, but intravenous iron administration is mandatory in patients with iron deficiency, especially if serum ferritin values are below 100 *μ*g/L [14]. Markers of inflammation should also be considered, and the optimal threshold for initiation of treatment, target hemoglobin, and doses of EPO should be standardized. The main question that requires an answer refers to the hemoglobin level to be achieved. There exists a risk of blood pressure increase at higher Hb levels, explained on increased viscosity and reduced nitric oxide availability. Blood transfusions should be considered in cases of severe anemia.

Iron deficiency, defined as depleted body iron stores (low serum hepcidin) and unmet cellular iron requirements (high-serum soluble transferrin receptor), is common in* acute heart failure* and is associated with poor outcome [39].

The simultaneous presence of anemia, heart failure, and chronic kidney diseases forms a pathological triangle called cardiorenal anemia syndrome [20]. The presence of even mild anemia and chronic kidney disease was associated with a synergistic amplification of the risk of death in patients with an old myocardial infarction, angina, heart failure, left ventricular hypertrophy, peripheral vascular disease, previous stroke, and thromboembolism [1, 40]. Chronic kidney disease causes anemia due to several mechanisms, including inadequate erythropoietin production, related to tubulointerstitial fibrosis and loss, and vascular obliteration [41].

**Figure 1:**
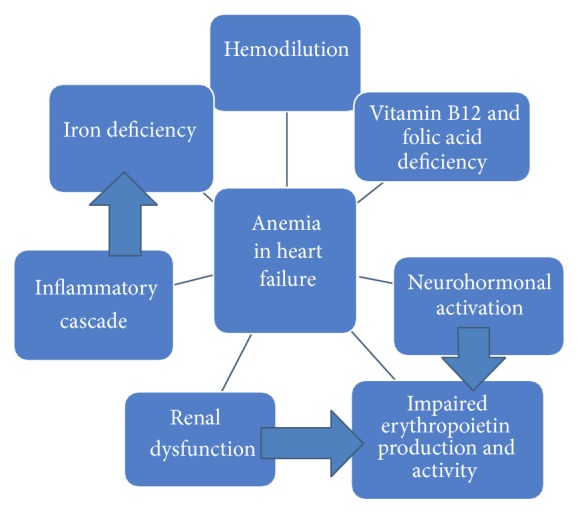
Causes of anemia in heart failure.

The main mechanisms of anemia in patients with heart failure are renal dysfunction, increased sympathetic and renin-angiotensin-aldosterone activity, hemodilution, absolute or functional iron deficiency, impaired erythropoietin production and activity, activation of the inflammatory cascade, angiotensin converting enzyme inhibition and angiotensin receptor blockade, and vitamin B12 and folic acid deficiency (Figure 1). Therapy includes iron, folic acid, blood transfusions, and erythropoiesis stimulating agents (Table 1). The main question still requiring an answer is the hemoglobin target level to be achieved in order to improve prognosis in heart failure.

### 2.2. Coronary Heart Disease

Anemia is a known risk factor for* ischemic heart disease* and a frequent finding in patients with acute coronary syndrome [42–44]. Multiple factors related to red blood cells are associated with coronary heart disease, including hemoglobin, hematocrit, RDW, and erytrocyte sedimentation rate [45]. Several studies suggest a detrimental effect of anemia in patients with acute myocardial infarction [41, 43, 46], related to reduced oxygen content in the blood, increased myocardial oxygen consumption due to elevated cardiac output to maintain appropriate tissue oxygenation, bleeding episodes during invasive procedures, anticoagulation, and inflammation (Table 2) [44, 47, 48]. A reduced oxygen transport capacity in anemia causes a compensatory increase of the heart rate, resulting in a shorter myocardial perfusion time in diastole [41]. A few studies in disease-free subjects and patients with vascular disease showed an association between increased hematocrit and increased risk of coronary heart disease, but low risk ratios were observed, and, therefore, the clinical usefulness of hematocrit alone is not clear [49]. Thrombotic events are important causes of morbidity and mortality in polycythemia vera [6]. Recent studies showed a negative correlation between hematocrit to blood viscosity ratio and likelihood of cardiac death in coronary heart disease patients [50].

The anemia of inflammation can reduce hemoglobin within 1-2 days, due to hemolysis of red blood cells and a suppression of the response to erythropoiesis mediated by tumor necrosis factor and acute changes in iron metabolism [48, 51, 52]. An increased uptake of iron in the reticuloendothelial system is responsible for the lowered Fe level, iron saturation of transferrin, and total iron binding capacity [52, 53]. A reverse association was found between C reactive protein and anemia, inflammation explaining the decline in hemoglobin [48].

The prevalence of anemia increases during hospitalization. Both the admission hemoglobin level and the subsequent fall in hemoglobin level >1.8 g/dL were associated with an increased risk of all-cause mortality or cardiogenic shock in patients with acute coronary syndrome [44]. The main causes of anemia were blood loss, hemodilution, kidney failure, and inflammatory reactions in response to myocardial injury [43, 44]. A modest fall in hemoglobin should have a beneficial effect due to reduction in blood viscosity, but greater falls increase myocardial ischemia and cause a neurohormonal reaction, which are responsible for the worse prognosis [44]. On the other hand, anemia at admission, especially associated with a history of bleeding, restricted the use of antithrombotic therapy, orienting toward a conservative therapy [44]. Hemoglobin is also an independent determinant of heart failure in acute coronary syndromes [54].

Sargento et al. evaluated the prognostic value of biohemorheological profile in transmural infarction survivors, revealing a close relationship between leukocyte count, protein C activity, and* erythrocyte membrane fluidity* and cardiovascular events during long term follow-up [4]. Membrane fluidity is an indicator of membrane microviscosity and lipid mobility and is influenced by anesthetics, antiarrhythmics, and insulin [55, 56].

The* red cell distribution width* (RDW), reflecting mean corpuscular volume heterogeneity, is an early parameter of iron deficiency, sideroblastic, vitamin B12, and folic acid deficiencies [21]. In patients with stable coronary artery disease, higher red cell distribution width (RDW), an index of anisocytosis, corresponds to higher comorbidity burdens (diabetes mellitus, heart failure, atrial fibrillation, peripheral vascular disease, and chronic kidney disease) and is an independent predictor of mortality [57]. The mentioned comorbidities are associated with a proinflammatory state and oxidative stress. Oxidative stress impairs membrane fluidity of the erythrocytes, reducing the life span of the red blood cells, and inflammation is known to block iron metabolism and erythropoietin response. Increased RDW is associated with impaired microvascular perfusion, causing hypoxia even in patients without anemia [57]. RDW was an independent predictor of death in patients with a previous myocardial infarction or stroke and of death secondary to cardiovascular diseases [58, 59].

Freudenberger and Carson investigated the relationship between* hemoglobin level* and adverse cardiovascular outcomes in women with chest pain, in the absence of myocardial infarction or congestive heart failure, reporting a higher risk of death from any causes, a higher risk of adverse outcomes, and shorter survival time free of adverse outcome, but no correlation between hemoglobin level and presence or severity of coronary atherosclerosis [60].

It has been suggested to correct anemia in patients with coronary artery disease, if hemoglobin levels have fallen to 9-10 g/dL in symptomatic angina [61]. In severe anemia, blood transfusions should be considered, but the data in regard to the effects of transfusions are contradictory. Wu et al. observed an increase in 30-day mortality in patients with acute coronary events after transfusions, and no impact if the* hematocrit* was less than 25% [62]. Other authors reported a beneficial effect of transfusions and an improved prognosis [63, 64]. The adverse effects of transfusions may be explained by the depletion of 2,3-diphosphoglyceric acid and nitric oxide stored in red blood cells, impairing oxygen release by hemoglobin and endothelial function [65]. Well designed, randomized, controlled trials of transfusion strategies are needed in order to provide guidelines with regard to blood transfusions in patients with acute coronary syndromes [49].

Components of the complete blood count, such as hematocrit, white blood cell count, and their subtypes, are associated with coronary heart disease and can improve our ability to predict coronary heart disease risk [49]. Several possible mechanisms of the role of red blood cells in coronary heart disease have been suggested, including viscosity, increased platelet aggregation associated with release of adenosine diphosphate, association with elevated serum cholesterol and triglycerides, deposition of cholesterol in the atherosclerotic plaque, stimulation of an excessive influx of macrophages, enlargement of the atherosclerotic necrotic core, and decreased fluidity of red blood cells [49].

**Figure 2:**
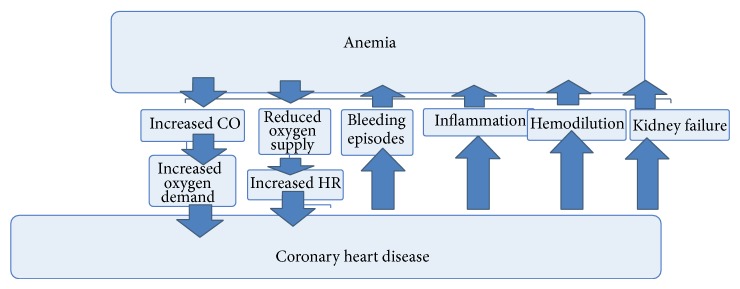
Links between anemia and coronary heart disease.

An imbalance between oxygen demand and supply, bleeding episodes due to invasive procedures and anticoagulation, inflammation, hemodilution, and kidney failure are the main mechanisms linking anemia and coronary heart disease (Figure 2). Red blood cell count, hemoglobin, hematocrit, and RDW should be monitored in patients with coronary heart disease.

### 2.3. Hypertension

Normocytic anemia is common among hypertensive patients. Lower hemoglobin concentrations were found in patients with uncontrolled than among those with well controlled hypertension, indicating a higher cardiovascular risk in uncontrolled hypertension [66]. Patients with anemia had higher nocturnal systolic and mean blood pressure and a tendency for increased diastolic blood pressure and lower dipping status compared to patients with normal hemoglobin levels [67, 68]. Leptin, the product of the human obesity gene, might be involved in the regulation of the rheologic behavior of erythrocytes and the microcirculation in hypertension [69].

Anemia is associated with higher cardiovascular risk, higher blood pressure values, and lower dipping status in hypertensive patients, and hemoglobin should be monitored in hypertensive patients.

### 2.4. Arrhythmias

Several electrocardiographic changes were described in patients with anemia, including ST segment depression, T wave inversion, QT interval prolongation, and reduced amplitude of the QRS complex [70–72]. A long ECG QT interval duration, exceeding 450 ms, is a predictor of ventricular arrhythmias and sudden cardiac death. The pathophysiological link between anemia and prolonged QT intervals is, probably, hypoxia and decreased myocardial oxygen supply. Anisocytosis, an early sign of anemia, and macrocytosis are also linked to prolonged QT intervals in hypertensive patients [73]. Positive correlations between serum ferritin or hemoglobin and QTc were observed in nonpregnant females with severe iron deficiency anemia [74].

Bindra et al. reported both supraventricular (sinus tachycardia, atrial premature contractions, and atrial fibrillation) and ventricular arrhythmias (ventricular premature contractions, ventricular tachycardia, and ventricular fibrillation) in patients with coronary heart disease and anemia [42]. Patients with lower levels of hemoglobin, iron, and total iron binding capacity were more likely to develop ventricular than supraventricular arrhythmias [42].

Prolonged QT intervals and arrhythmia risk are linked to anemia, macrocytosis, anisocytosis, serum ferritin, and hemoglobin, and hypoxemia supports these links.

### 2.5. Stroke

Stroke is the leading cause of adult disability and the third cause of mortality and has a high prevalence, considering the growth and aging of the population. Complete blood count abnormalities represent a useful tool for stroke patients' prognosis (Table 3). A low hematocrit means hypoxia and cerebral ischemia. Blood flow augmentation and turbulence due to anemia enables migration of a thrombus and embolism [75]. Anemia was associated with an increased mortality after ischemic and hemorrhagic stroke [76, 77]. Cardiac surgery patients, routinely hemodiluted, often sustain perioperative cerebral infarction. At hemoglobin concentrations of of 10–12 g%, increased cerebral blood flow and oxygen extraction are sufficient to enable penumbra oxygen uptake to remain nearly normal [78]. Penumbra oxygen extraction reserves are nearly exhausted even after moderate anemia [79]. Several previous studies focused on the impact of a high hematocrit level on stroke and reported a higher stroke prevalence related to a high hematocrit level [80], because of its contribution to cerebrovascular blood viscosity and its potential role in cerebral atherogenesis [81, 82]. Diamond et al. found midrange hematocrit levels (45%) as having the best outcome in stroke patients [83].

Anemia enables cerebral ischemia, blood flow turbulence, migration of a thrombus, and embolism and reduces penumbra oxygen reserves. Besides that, anemia was associated with increased mortality after ischemic and hemorrhagic stroke.

## 3. Cardiovascular Consequences of Hereditary Forms of Anemia

### 3.1. Cardiovascular Consequences of Thalassemia

Thalassemia is the most common hereditary disease, a genetic blood disorder due to reduced synthesis of beta or alpha globin chains [84, 85]. Regular transfusion therapy improves the quality of life but also causes iron overload [86]. Transfusion iron overload can directly affect the heart tissue through iron deposition in the ventricular walls, causing left ventricular systolic and diastolic dysfunction (later signs of iron overload), pulmonary hypertension, valvulopathies, arrhythmias, and pericarditis [85, 87]. Congestive heart failure is the leading cause of morbidity and mortality, but sudden cardiac death may also occur, even in the absence of cardiac dysfunction [84]. The degree of cardiac dysfunction depends on the quantity of iron deposited in the myocardial fibers and the number of the affected fibers [87]. The iron is stored in intracellular lysosomes as nontoxic ferritin and hemosiderin, but above certain concentrations, reactive iron species are generated and the cell begins to fail [87]. The patchy nature of cardiac iron deposition may provide substrates for reentry and risk of fatal arrhythmias in patients with* beta-thalassemia*, which explains the appearance of premature ventricular contractions, ventricular tachycardia, and late ventricular potentials [88, 89]. Iron toxicity arrhythmias are often automatic, represented by polymorphic atrial and ventricular arrhythmias [86]. Bradicardia and repolarization abnormalities on 12-lead electrocardiography including QT interval prolongation, leftward shift of the T wave axis, and generalized STT changes are the most specific markers for iron cardiomyopathy in thalassemia major and may be helpful to stratify cardiac risk when cardiac MRI is unavailable [84]. Tachycardia, as physiologic compensation of anemia, strongly associated with vascular inflammation, QT prolongation, STT abnormalities and intraventricular conductions delays due to ventricular dilation, were also reported in thalassemia major, regardless of cardiac iron status [84]. The absolute QT interval duration was a better marker of cardiac iron than heart rate corrected QT interval because the interaction between heart rate and QT interval is impaired due to iron overload [84]. Impairment of delayed rectifier potassium channel and calcium channels may explain the changes in repolarization [84, 86]. ECG recording could be used by hematologists as a screening tool in those patients. Iron deposition, microvascular scarring combined with inflammatory and immunogenetic factors, endocrine deficiencies, chronically elevated cardiac output secondary to anemia, increased cardiac afterload due to accelerated vascular aging, and the hypercoagulability state are involved in the pathophysiology of cardiac dysfunction in beta-thalassemia major [84, 85, 90, 91]. Within cells, iron catalyses the production of reactive oxygen species, causing lipid peroxidation and organelle damage [85]. Atrial mechanical depression was reported as a very early sign of cardiac damage in beta-thalassemia, prior to diastolic and systolic left ventricular dysfunction [91]. Males and females are at the same risk of accumulating iron in their hearts, but women better tolerate iron toxicity, probably due to a more effective antioxidant defense, a slower metabolic rate, more active immune function, and reduction in the activity of growth hormone [92]. The cardiomyopathy may be reversible if iron chelation therapy is given [87]. Thalassemia major patients with diabetes mellitus had a higher risk of cardiac complications, including heart failure, hyperkinetic arrhythmias, and myocardial fibrosis [93]. A protective effect of beta-thalassemia against myocardial infarction was demonstrated in thalassemia minor due to a favorable lipidemic profile, a better cardiovascular risk factor, and 24 h blood pressure profile, compared with anemic and nonanemic hypertensives [94, 95].

The survival of patients with thalassemia major has improved lately, as a result of regular transfusions and chelation therapy and new imaging methods, which allow better management of iron overload [92]. There also exists an increased risk of thrombosis in beta-thalassemia intermedia and major, causing deep venous thrombosis, pulmonary hypertension, and pulmonary embolism, inversely correlated with the hemoglobin level [87]. Several biological risk factors for thrombosis were mentioned, including splenectomy, increased levels of thrombin-antithrombin III complex, red cell phosphatidylserine exposure, and plasma coagulation factor abnormalities [87].

The main cardiovascular effects of thalassemia are due to hypoxia and iron accumulation. Iron overload explains most cardiovascular problems in thalassemic patients, such as left ventricular systolic and diastolic dysfunction, reentry ventricular arrhythmias, atrial mechanical depression, myocardial fibrosis, vascular inflammation with early vascular aging, and hypercoagulability.

### 3.2. Cardiovascular Consequences of Sickle Cell Anemia

Sickle cell anemia is caused by hemoglobin S, which changes the shape of the red blood cells from discs to sickles, reduces their deformability, and enhances stickiness, leading to obstructive adhesion of sickle cells [96]. Reperfusion injuries and endothelial cell damages result. Obstruction and inflammation cause further hypoxia, acidosis and sickling [96]. Cardiovascular abnormalities are common in sickle cell anemia, including cardiac enlargement, myocardial infarction, acute stroke, chronic cerebral ischemia, arrhythmias, increased arterial stiffness, and microcirculation damage due to vasoocclusive crisis, QT interval borderline or moderate prolongation, and cardiac autonomic neuropathy [78, 96–101]. Autonomic dysfunction and QT interval duration were correlated in patients with sickle cell anemia, suggesting significant clinical implications. The presence of hypoxia due to chronic anemia and cardiac autonomic neuropathy, with unopposed sympathetic activation and abnormalities of ventricular repolarization, predisposes to ventricular electrical instability and increased arrhythmia risk [100]. Several foci of old and new degeneration in the sinus and atrioventricular node and His bundle, fibrosis, and fibromuscular dysplasia affecting small coronary arteries were revealed by postmortem studies in patients with sickle cell anemia, responsible for electrical instability [102]. Several other electrocardiographic abnormalities were described in patients with sickle cell cardiovascular autonomic neuropathy, including increased P wave duration, RR interval, and QTc dispersion, increased frequencies of Q waves, and first degree atrioventricular block [103]. Only increased tricuspid regurgitant jet velocity was significantly associated with QT interval among patients with S hemoglobinosis [78]. QTc prolongation was not associated with left ventricular hypertrophy in sickle cell anemia, and elevated pulmonary pressure, hemolysis, and acute chest syndrome may represent risk factors for prolonged QTc [99]. QTc dispersion, the difference between maximal and minimal QT interval duration in the 12 standard ECG leads, was also significantly increased in sickle cell disease, especially in patients with pulmonary hypertension, correlated with regional inhomogeneity of ventricular repolarization [104]. Nitric oxide scavenging by free hemoglobin is implicated in the pulmonary artery disease of sickle cell anemia [87].

Meloni et al. compared biventricular dimensions and function using cardiovascular magnetic resonance in pediatric, chronically transfused patients with sickle cell disease and thalassemia major and normal cardiac iron levels, reporting significantly greater biventricular dilation and left ventricular hypertrophy and lower left ventricular ejection fraction in the first group [105].

Tantawy et al. investigated 50 young patients with sickle cell disease, revealing hypercoagulability, significantly higher aortic stiffness, and pulmonary artery pressure compared to healthy controls [101]. The hypercoagulability may result from chronic hemolysis and circulating cell-derived microparticles originating from activated platelets and erythrocytes [101]. The cell-derived particles can be considered as potential biological biomarkers for vascular dysfunction and disease severity, being significantly increased in sickle cell disease patients with pulmonary hypertension, sickling crises, acute chest syndrome, stroke, history of thrombosis, or splenectomy, being positively correlated with aortic stiffness, pulmonary artery pressure, and tricuspid regurgitant velocity, and being negatively correlated with aortic distensibility [101].

The main cardiovascular effects of sickle cell disease are due to the rigid erythrocytes with hemoglobin S, impairing blood flow, enabling vasoocclusive crises, and explaining the appearance of myocardial infarction, stroke, microcirculation damage, and degeneration of the excitoconductor system. Sickle cell anemia is also associated with other cardiovascular complications, such as cardiac autonomic neuropathy, arrhythmias, tricuspid regurgitation, and increased arterial stiffness. Besides disordered hemoglobin structure and function, a prothrombotic state due to changes in the hemostatic system, such as thrombin activation, decreased levels of anticoagulants, impaired fibrinolysis, and platelet activation are also involved in the pathogenesis of sickle cell disease [106].

### 3.3. Cardiovascular Consequences of Hereditary Spherocytosis

Hereditary spherocytosis is the most common inherited hemolytic anemia [107], characterized by the presence of spherical-shaped erythrocytes on the peripheral blood smear, with osmotic fragility, due to abnormalities of various erythrocyte membrane proteins, especially spectrin and ankyrin [108]. Hemolytic, aplastic, and megaloblastic crises may occur [108]. Aplastic crises appear after virally induced bone marrow suppression and may lead to severe anemia with serious complications such as congestive heart failure [108]. Chronic anemia is well tolerated by children, and it can rarely lead to increased cardiac output, cardiomegaly, and leg ulcers [109].

Splenectomy is recommended in patients with moderate and severe forms, considering that it results in near complete resolution of hemolysis [107]. Persons without a functional spleen, with hereditary or chronic hemolytic anemia, and even without hematological conditions are at increased risk of atherothrombosis, deep vein, portal and superior mesenteric vein thrombosis, and pulmonary arterial hypertension [107, 110, 111], due to the prothrombotic state caused by higher thrombin formation [110]. Components of the erythrocyte membrane facilitate coagulation and the loss of the filtering function of the spleen allows abnormal red blood cells to remain in the peripheral circulation, enabling the activation of the coagulation cascade, especially in chronic hemolysis [110]. A prospective, cross-sectional study including patients with hereditary spherocytosis who underwent splenectomy revealed higher LDL-cholesterol, fibrinogen, and homocysteine values, indicating a higher cardiovascular risk [107].

Anticoagulation prophylaxis and lipid lowering drugs should be considered after splenectomy in patients with chronic hemolytic disorders, besides postsplenectomy sepsis prophylaxis, especially if there is an additional high thromboembolic risk due to surgery or immobilisation [111].

## 4. Conclusions

The presence of anemia is associated with a special risk for patients with any form of proatherosclerotic condition and heart disease. Anemia became a new therapeutic target in patients with cardiovascular pathology, improving oxygen supply. Guidelines must be updated for the management of patients with anemia and cardiovascular diseases, and targets for hemoglobin level should be established, in order to improve prognosis. Early primary care diagnosis, monitoring, and management of patients with anemia and cardiovascular morbidity and evaluation of iron, vitamin B12, folic acid, and nutritional status may be worthwhile. On the other hand, the unfavorable effects of therapy should also be considered, including blood pressure elevation, the prothrombotic effect, and increased viscosity. Risk scores in several cardiovascular diseases should include red blood cell count and RDW.

Complete blood count abnormalities and hemorheological parameters represent useful, inexpensive, widely available tools for the management and prognosis of patients with coronary heart disease, heart failure, hypertension, arrhythmias, and stroke.

The main cardiovascular effects of sickle cell disease and thalassemia are due to hypoxia and iron accumulation, respectively. Thromboembolic risk and lipid profile should be monitored after splenectomy in patients with congenital chronic hemolytic anemia.

## Figures and Tables

**Table 1:** Heart failure (HF) and anemia.

Number of patients	Findings	Reference
48,612	A higher prevalence of low hemoglobin in hospitalized patients than noted in randomized HF trials Lower Hb is associated with higher morbidity and mortality in hospitalized patients with HF	Young et al., 2008 [12]

5477	In patients with complicated acute myocardial infarction, anemia on admission and/or reductions in hemoglobin are independent risk factors for mortality and hospitalization	Anker et al., 2009 [14]

528	Anemia was more prevalent in patients with preserved left ventricular ejection fraction (LVEF) than in those with reduced LVEF	Berry et al., 2005 [16]

137	Anemia is common in patients with heart failure and a normal ejection fraction and is associated with greater elevations in serum B-type natriuretic peptide, more severe diastolic dysfunction, and a worse prognosis	Brucks et al., 2004 [17]

20	Chronic heart failure is associated with profound and general bone marrow dysfunction	Westenbrink et al., 2010 [18]

165	Iron deficiency is common in acute heart failure and identifies those with a poor outcome	Jankowska et al., 2013 [21], Jankowska et al., 2014 [38]

4	In patients with edema caused by severe anemia there is salt and water retention, reduction of renal blood flow and glomerular filtration rate, and neurohormonal activation. Patients with anemia have a high cardiac output, a low systemic vascular resistance, and blood pressure. The low concentration of hemoglobin causes a reduced inhibition of basal endothelium-derived relaxing factor activity and leads to generalised vasodilation	Anand et al., 1993 [25]

32	Therapy of anemia in congestive heart failure with erythropoietin and intravenous iron improves cardiac and renal function and reduces hospitalization and the need for diuretics	Silverberg et al., 2001 [27]

26	Erythropoietin significantly increases exercise capacity in patients with chronic heart failure. One mechanism of improvement in peak oxygen consumption is increased oxygen delivery from increased hemoglobin concentration	Mancini et al., 2003 [29]

40	In anemic chronic heart failure patients, correction of anemia with erythropoietin and oral iron improves the NYHA status, measured exercise endurance, oxygen use during exercise, renal function, and plasma B-type natriuretic peptide levels and reduces the need for hospitalization	Palazzuoli et al., 2006 [30]

160	Treatment with darbepoetin alfa in patients with chronic heart failure and anemia raised Hb and improved some quality of life indices	van Veldhuisen et al., 2007 [31]

1432	The use of a target hemoglobin of 13.5 per deciliter (as compared with 11.3 g per deciliter) was associated with increased risk of death, hospitalizations for congestive heart failure and myocardial infarction, and no improvement in the quality of life	Singh et al., 2006 [33]

603	Early complete correction of anemia does not reduce the risk of cardiovascular events in patients with chronic kideny disease	Drüeke et al., 2006 [34]

40	Intravenous iron therapy substantially reduced NT-proBNP and inflammatory status in anemic patients with chronic heart failure and moderate chronic renal failure, improving left ventricular ejection fraction, NYHA functional class, exercise capacity, renal function, and quality of life	Toblli et al., 2007 [36]

32	Intravenous iron causes a marked increase in hemoglobin in anemic congestive heart failure patients, associated with improved cardiac remodeling and NYHA classification	Usmanov et al., 2008 [37]

**Table 2:** Coronary heart disease and anemia.

Number of patients	Findings	Reference
417	Anemia is a significant risk factor in ischemic heart disease (IHD), and it correlates with advanced IHD, chronic heart failure, rhythm distrurbance, and higher mortality rate	Zeidman et al., 2004 [41]

320	Abnormal hemoglobin levels are common in acute coronary syndromes. Anemia was associated with increasing age, interventional management, and adverse in-hospital outcomes	Bindra et al., 2006 [42]

542	In high-risk acute coronary syndrome patients both the admission hemoglobin level and subsequent fall in hemoglobin level >1.8 g/dL were associated with an increased risk of all-cause mortality or cardiogenic shock	González-Ferrer et al., 2008 [43]

1,497	Anemia on admission in patients with acute myocardial infarction treated in the acute phase with percutaneous coronary intervention is associated with increased mortality, especially in the subgroups with incomplete revascularization and multivessel disease	Kurek et al., 2010 [45]

32,170	A low baseline hemoglobin level is an independent predictor of the risk of major bleeding and death in acute coronary syndromes	Bassand et al., 2010 [46]

1017	Inflammation-sensitive proteins are associated with lower hemoglobin concentrations in acute myocardial infarction patients	Steinvil et al., 2012 [47]

109	Low hematocrit/blood viscosity ratio can be regarded as a risk factor of cardiac death in coronary heart disease	Kenyeres et al., 2008 [49]

56	There was a significant reduction of plasma iron, total iron binding capacity, and plasma transferrin and a significant elevation of serum ferritin after myocardial infarction, changes, probably influenced by the extent of tissue necrosis	Griffiths et al., 1985 [50]

84	Serum ferritin and iron levels are increased after a myocardial infarction due to the traumatic effect of the infarction. An increased uptake of iron in the reticuloendothelial system for synthesis of ferritin may account for the lowered serum iron level and the iron saturation of transferrin	van der Schouw et al., 1990 [51]

2,036	Total iron binding capacity is an independent negative risk factor for myocardial infarction	Magnusson et al., 1994 [52]

2,310	Anemia is a common comorbidity in patients with acute coronary syndromes and a powerful independent determinant of left ventricular failure	Archbold et al., 2006 [53]

2,550	Higher red cell distribution width (RDW) values correspond to higher comorbidity burdens and higher mortality in patients with stable coronary artery disease	Osadnik et al., 2013 [56]

4,111	A graded independent relation was found between higher levels of red cell distribution width and the risk of death and cardiovascular events in people with prior myocardial infarction	Tonelli et al., 2008 [57]

936	Lower hemoglobin levels were linked with higher risk for adverse outcomes in women with suspected ischemia in the absence of acute myocardial infarction or congestive heart failure	Arant et al., 2004 [59]

24,112	Blood transfusion in patients with acute coronary syndromes is associated with higher mortality	Rao et al., 2004 [61]

78,974	Blood transfusion is associated with a lower short-term mortality rate among elderly patients with acute myocardial infarction and a hematocrit at admission of 30%	Wu et al., 2001 [62]

39,922	Anemia is a powerful and independent predictor of major cardiovascular events in patients with acute coronary syndromes	Sabatine et al., 2005 [63]

**Table 3:** Anemia and stroke.

Number of patients	Findings	Reference
480	Elevated red cell distribution width is associated with stroke occurrence and strongly predicts both cardiovascular and all-cause deaths in persons with known stroke	Ani and Ovbiagele, 2009 [58]

16	Bleeding and subsequent anemia may precipitate atherothrombotic cerebral infarction	Kim and Kang, 2000 [75]

774	A higher mortality rate was found in stroke patients with anemia and the stroke risk factors of being older than 70 years and having chronic renal failure were more prevalent	Huang et al., 2009 [76]

484	Anemia independently predicted mortality at 6 months and 1 year after the initial episode of intracerebral hemorrhage	Zeng et al., 2014 [77]

3,481	High hematocrit may represent in women an independent predictor of mortality after ischemic stroke	Sacco et al., 2007 [80]

5,185	Risk of stroke was proportional to the blood hemoglobin concentration	Kannel et al., 1972 [81]

6	A hypercirculatory state in patients with sickle cell disease, accompanied by anemia and abnormal red cells, may make patients particularly prone to ischemic infarction	Herold et al., 1986 [82]

1,012	An association exists between hematocrit level at the time of ischemic stroke and discharge outcome	Diamond et al., 2003 [83]
